# Patients’ perspective of disease and medication adherence for type 2 diabetes in an urban area in Bangladesh: a qualitative study

**DOI:** 10.1186/s13104-017-2454-7

**Published:** 2017-03-21

**Authors:** Sheikh Mohammed Shariful Islam, Tuhin Biswas, Faiz A. Bhuiyan, Kamrun Mustafa, Anwar Islam

**Affiliations:** 1 NCD Program, International Center for Diarrhoeal Disease Research, Bangladesh (ICDDR,B), 68, Shaheed Tajuddin Ahmed Sarani, Mohakhali, Dhaka, 1212 Bangladesh; 2 Center for International Health, Ludwig-Maximilians Univetsitat, Munich, Germany; 3 Department of Public Health, The University of Southern Mississippi, Hattiesburg, MS USA; 4 Bangladesh University of Health Science (BUHS), Dhaka, Bangladesh; 5 Faculty of Health, School of Health Policy and Management, York University, Toronto, Canada; 6 Department of Public Health, North South University, Dhaka, Bangladesh; 7 The George Institute for Global Health, University of Sydney, Sydney, Australia

**Keywords:** Type 2 diabetes, Medication adherence, Compliance, Perception, Glycemic control, Bangladesh

## Abstract

**Background:**

Patients’ perspective of diabetes and adherence to its prescribed medications is a significant predictor of glycemic control and overall management of the disease. However, there is a paucity of such information in Bangladesh. This study aimed to explore patients’ perspective of diabetes, their experience of taking oral hypoglycemic medications and explore factors that contribute to medication adherence in patients with type 2 diabetes in Bangladesh.

**Methods:**

We conducted in-depth face-to-face interviews with 12 type 2 diabetes patients attending a tertiary hospital in Dhaka city between February and March, 2014. Participants were purposively sampled representing different age groups, education levels, years since diagnosis with diabetes, and glycemic status, to achieve maximum variation sampling. All interviews were conducted using a topic guide and were audio-recorded, transcribed verbatim, checked for errors, coded and analyzed by means of a qualitative content analysis framework.

**Results:**

The data analysis generated rich information on the participants’ knowledge and perception on diabetes, its causes, self-management, medication use, adverse effects of medication use, medication adherence, and impact of diabetes, Although most of the participants demonstrated substantive knowledge on diabetes and its consequences, they also reported numerous misconceptions about the disease. Knowledge on diabetes medication, their appropriate use and side effects was rather poor. Respondents also reported non-compliance to dietary and physical activity advice by their physicians and concerns on diabetes diabetes-induced psychological stress. High cost of medications, concerns over medication side effects and forgetfulness was noted as factors for non-adherence to medication.

**Conclusion:**

Participants’ knowledge and perception on diabetes are key factors determining their adherence to medications and, thereby, diabetes management. Healthcare providers should explore to better understand patients’ perspective on diabetes, medication beliefs, identify psychological stress and provide more effective health education interventions to enhance medication adherence.

## Background

 In recent decades, the prevalence of non-communicable diseases (NCDs) has increased significantly worldwide, especially in developing countries, where NCDs currently account for the highest burden of morbidity and mortality [1, 2]. The NCD epidemic in low and middle income countries is considered as a result of the rural–urban migration, rapid urbanization, low awareness and life style related factors such as sedentary lifestyle, and unhealthy food habit, which are mostly preventable [3]. The rapid rise of NCDs, such as diabetes is an alarming public health problem for Bangladesh [4, 5]. A recent study using data from the 2011 Bangladesh Demography and Health Survey, reported that the age-adjusted prevalence of diabetes and prediabetes in Bangladesh is 9.7 and 22.4%, respectively [6]. As projected by the International Diabetes Federation, by 2030 Bangladesh would emerge as one of the countries with the largest number of people with type 2 diabetes [7].

Diabetes is a serious disease that imposes heavy financial burden in affected individuals, their families and the nation at large [8, 9]. A recent study conducted on patients with type 2 diabetes attending an urban clinic in the capital city of Dhaka found that almost two-third of the patients had uncontrolled diabetes [10]. Most of these patients were newly diagnosed cases with several co-morbidity. Another study reported poor adherence to lifestyle and medication among patients with type 2 diabetes in Bangladesh resulting in overall poor quality of life [11]. Research evidence suggests that adherence to medications and lifestyle modifications have significant impact on the outcome of diabetes treatment and care [12–14]. However, non-adherence to medication for diabetes remains an unresolved problem, which can lead to several costly and life threatening complication and patient’s perspective are often neglected in treatment design. Consequently, it is imperative to understand patients’ perspective on diabetes, its medications and the importance of adherence to medications for glycemic control in order to promote effective and optimum diabetes care. Unfortunately, Bangladesh lacks credible research and data on patients’ perspective on diabetes, its treatment and factors associated with medication adherence [15, 16]. The present study, therefore, was designed to address some of these paucity of data issues and aimed at better understanding patients’ perspective on type 2 diabetes and factors underscoring their adherence to diabetes medications in an urban area in Bangladesh.

## Methods

### Study design, location and participants

We conducted a qualitative study based on in-depth interviews with patients with type 2 diabetes attending the outpatient department of the Bangladesh Institute of Health Science (BIHS) hospital in Dhaka, Bangladesh between February and March, 2014. As our primary objective was to understand medication adherence from the patients’ perspective, we adopted this methodological approach, which allows a flexible exploration of respondents’ experience and attitudes [17, 18]. The BIHS hospital is affiliated with the Diabetes Association of Bangladesh (DAB)—the prime agency providing comprehensive diabetes care throughout the country using specialized clinics and hospitals [19]. The outpatient department of BIHS hospital serves a large number of diabetes patients in Dhaka city and surrounding areas. The BIHS hospital was purposefully chosen as it represents the urban population with diabetes in and around Dhaka city. Interviews were conducted within the hospital setting in private consultation rooms that ensured adequate privacy for both the interviewers and interviewees.

The study participants were purposively selected to gather information-rich cases, based on the following inclusion criteria: (a) patients diagnosed with type 2 diabetes by the BIHS attending physician according to WHO criteria; (b) Currently on oral hypoglycemic medication based on the prescription by the attending physician, (c) diagnosed with type 2 diabetes for at least one year, and (d) registered with the BIHS Hospital. We excluded participants on insulin therapy, those with type 1 diabetes, gestational diabetes or having other serious co-morbidities requiring immediate care. We initially approached face-to-face 20 participants based on our selection criteria to gather rich and thick description. None of the participants refused to participate. We scheduled the interviews at the BIHS hospital when the participants came for follow up with their physician. We interviewed 12 participants in the study until no new data were obtained and saturation point was reached [20].

### Data collection

A semi-structured interview guideline (Table 1) was developed by the principal researcher following extensive literature review and considering the perspective of patients regarding medication adherence and glycemic control. The interview guide was developed in English and translated into Bengali using standards methods of translation and back-translation. The interview guide was pilot tested on two participants with type 2 diabetes at a diabetes center in Dhaka city and modified based on the feedback of the participants and the interviewer. The interviews were conducted by a trained Anthropologist (FAB) with expertise and experience in conducting qualitative research in institutional as well as in community settings with the support of a research officer (TB) experienced in hospital data collection. The data collection team were trained by the principal investigator and senior scientists at the Center for Control of Chronic Diseases (CCCD) within the International Center for Diarrhoeal Diseases Research, Bangladesh (ICDDR,B) for two weeks on the study protocol, objectives of the study, research ethics and data collection methods. On average, an interview took approximately 40–45 min to be completed. However, one interview took substantially more time as it had to be re-scheduled due to time constraint of the interviewee. The interviewer used a short structured questionnaire to collect demographic and clinical data on glycated hemoglobin (HbA1c) and use of medications by the participants and through review of patients’ medical records at the outset of the interview. During the interview, the Research Officer took written notes and made audio recordings with permission of the respondents. The Research Officer also checked the sequential responses of the interviewees with a view to ensure full completion of the questionnaire.

**Table 1:** In-depth interview guide

Category	Questions
Knowledge about diabetes	What do you know about diabetes?
	What causes diabetes? (Probe: Ask about genetics, food habits, physical exercise, sugar)
	What is the impact of diabetes on your life?
	What do you think are the complications of diabetes?
	What can you do to control your diabetes?
	What prevents you from managing your diabetes?
Medication use and adherence	Tell me about all the medicines that you are taking currently. (Probe: names, doses, and timings)
	How do you feel to take all the medicines as advised by your physician? (Probe: emotional issues)
	Do you know about the importance of taking all your medicines? (Probe: why is it important or not?)
	What do you know about the side effects of your medicines?
	What problems do you face to take your medicines?
	Who pays for your medicines?
	How much support do you get from your family and friends about taking all your medicines as advised by your physician?

The study was focused on the following interdependent and interrelated issues: the participants’ knowledge on and perception about diabetes and its long-term health impact; their views on diabetes treatment and care and the importance of adherence to medications. With a view to generate more meaningful information, the respondents were asked probing questions and were allowed flexibility of time to reflect on questions and respond accordingly. During the interviews, the research officer took written notes, made audio recordings with permission of the respondents and performed member checking [21]. Moreover, at the end of the interview session, respondents were allowed to add additional points as they deem fit.

### Data analysis

All interviews were audio-recorded and transcribed verbatim. Research investigators checked the data quality and identified the gaps. The field teams were immediately asked to address these identified gaps. We performed forward and backward translation of the interview guide and all transcriptions (n = 12) from Bengali to English following ISPOR Principles of Good Practice:  The Cross-Cultural Adaptation Process for Patient-Reported Outcomes Measures was applied to ensure consistency and congruity of all data. These measures included the following steps: 1. Preparation; 2. Forward Translation; 3. Reconciliation; 4. Back Translation; 5. Back Translation Review; 6. Harmonization; 7. Cognitive Debriefing; 8. Review of Cognitive Debriefing Results and Finalization; 9. Proofreading; and 10. Final Report [22]. Transcribed interviews were analyzed by qualitative content analysis framework [23, 24]. To ensure validity and reliability of the analyses, the interview transcripts were separately and independently coded by two researchers. The codes were compared by the two researchers and any differences were resolved by discussion.

## Results

A total of 12 patients (5 males and 7 females) diagnosed with type 2 diabetes, aged between 38 and 66 years (mean age 52.2 years) participated in this study. The gender balance represents the higher number of female patients as reported in previous study in the same location [10]. While three participants each had completed primary and secondary level education, five of them had completed university level education, and only one was illiterate. The number of years diagnosed with diabetes ranged from 1 to 18 years. The number of total medications prescribed for the participants per day ranged from 3 to 6, with a median of 4 medicines per day. Nine participants reported poor glycemic control (HbA1c ≥ 7%). The characteristics of the respondents are presented in Table 2 and the key themes in Table 3.

**Table 2:** Characteristics of the study participants

Description	N
Sex	
Male	5
Female	7
Education	
Illiterate	1
Primary	3
Secondary	3
University	5
Diabetes duration (years)	
1–3	3
4–6	4
More than 6 years	5
Number of medication	
3	2
4	5
5	2
6	3
Glycemic status	
HbA1c < 7% (controlled)	3
HbA1c ≥ 7% (uncontrolled)	9

**Table 3:** Key themes on patients’ perspective of diabetes and medication adherence

	Key themes
Knowledge about diabetes	Knowledge about cause of diabetes and its effects Poor self-management Misconception that mild diabetes does not require medication Lack of compliance on diet and physical activity Psychological stress
Medication use and adherence	Lack of education on diabetes medication Forgetfulness to take medication High cost of medication Lack of knowledge on medication side effects

### Knowledge and perceptions about diabetes

Participants were asked about their knowledge and perceptions about diabetes, its causes, long-term impact and on how diabetes may be controlled.

All participants were familiar with the term “diabetes”. Participants used the term “border line” or “mild diabetes” to describe a condition when “sugar in urine” is present which is evidenced by observing after passing urine. “After urination if a person has mild diabetes ants will gather near the place of urination (M3_48 years).” Two participants expressed the opinion that there was no need to go on medication for mild diabetes and that dietary control alone would suffice (M5_44 years, F2_38 years). A number of participants equated “sugar in blood” with the onset of diabetes. However, most considered diabetes as a serious chronic condition requiring lifelong treatment and medications. “Being borderline is not so bad, but diabetes is scary, because of all the damages it causes to the body (F2_38 years).” Participants considered diabetes to be a very common condition in Bangladesh. For example, one participant commented: “Now-a-days in almost every house you will find a person with diabetes (F4_49 years).”

A few respondents also commented on embarrassing situations often encountered by diabetic patients. Patients with diabetes need to use wash rooms frequently and such facilities are not available in many outdoor locations in Bangladesh. Thus, diabetes patients often face difficulties moving outdoors or restricted from traveling or visiting other places.

### Causes of diabetes

Most of the respondent believed that the primary cause of diabetes and poor glycemic control was too much sugar in the diet. Other etiological factors mentioned by the informants included heredity, sedentary lifestyle and obesity. Some respondents identified physical or psychological stress as a perceived cause of diabetes. Several participants noted the consumption of too much sweets and rice as contributory factor for diabetes. For many participants their understanding of diabetes originated with their family experience with the illness as stated by a participant, “It runs in the family. My parents had it as well as my two brothers. I knew that eventually I would get it too (F1_48 years).”

A few participants considered the growth of the fast food industry and the increasing use of chemicals in food as factors underlying the rapid rise in the prevalence of diabetes. “Earlier foods were pure and now the use of excess chemicals and adulterated food causes many diseases including diabetes (F3_51 years).” Another participant noted: “I used to drink coca-cola every day for fifteen years, which is bad for health and might have caused my diabetes (F5_61 years).”

### Impact of diabetes

Most of the respondents were aware that diabetes could damage some of the vital organs of human body. Feeling weak, dryness in the mouth and a frequent sense of hunger were some of the other complications of diabetes mentioned noted by the respondents. In short, an overwhelming majority of the respondents could list at least two complications associated with diabetes, most of them were also aware of the role of heredity in this regard. For example, one of the respondents reported, “My mother was a diabetic for 26 years. She took insulin regularly but still had eye problems, heart disease and died of kidney failure. I am suffering from diabetes for 12 years and taking oral medicines, but later I will also need insulin and develop heart and kidney problems (F3_51 years)”.

Along with physical impact, diabetes also has psychological and social impacts on its victims. Psychological stress associated with diabetes often negatively affect the patient’s ability to maintain the rigors of recommended treatment and care. Respondents often lamented on the restrictions imposed by diabetes on their food habits. For example, one of the male respondents commented: “I liked to eat sweets very much, but after being identified as a patient with diabetes, I have to reduce the amount of sweets in my diet. I don’t have any wish to eat sweet anymore. (M1_59 years).” Many felt that diabetes-induced psychological stress often make them behave negatively demonstrating anger and frustration with family members, neighbors and other acquaintances, such as rickshaw pullers and street vendors. This may also often lead to physical violence against spouse or children. Expressing his frustration on the sudden onset of diabetes, one of the respondents remarked: “It’s frustrating, that there are no cures and you have to take medicines life-long and visit the doctors (M2_53 years).”

### Self-management of diabetes

For most of the respondents, diet, medication and exercise are the three most important components for self-management of diabetes. While they noted that that diet and medications could reduce the blood sugar, for most exercise remains a missing ingredient. Most of them could not engage in exercise or physical activity due to lack of lack of time and suitable place for such activity. One participant mentioned that “Well, I would like to walk every day for more than 30 min, but the roads in my area are not suitable for walking, there is no walkway or park nearby and I am ashamed of doing any exercise in my home (F6_52 years).” Another participant said, “I tried walking every day for three months, but it did not help to control my disease and weight. Now, I have stopped walking and do little exercise at home (F2_38 years).”

Most of the respondents agreed on the importance of following the dietary plan recommended by their physician and/or dietician. However, most of them did not follow or adhered to the dietary plan. Most of the participants stated that they felt depressed to follow dietary restrictions. Some of them were not happy with the amount and frequency of food intake recommended by the doctor. One of the male respondents mentioned, “I need to eat small amounts of food frequently, but in a work place who will provide food so frequently? (M2_48 years)”. A few participants noted that they were not able to control their diet, especially during the summer season and took mangoes and other fruits without much restriction. Two participants reported that fruits were not harmful for diabetes.

Though many of the respondents stated that a meal without rice did not seem complete, many of them replaced rice with ‘*rooti* or bread’. In the same way, sweet dishes were replaced with bitter foods or vegetables such as immature banana or papaya, bitter gourd, juice of green *Chirata* and *neem* leaf.

### Medication use and its effects

The study indicates that there is a significant gap between patients’ knowledge of and adherence to medications. For example, only two participants could properly list all the medications prescribed for them, the exact doses and time of intake. One of the respondents said, “I have diabetes, I take five different medicines, but I am not sure which ones are specifically for diabetes and the dose of each medicine (M1_59 years).” Respondents with higher levels of education were able to list the name of the medicines they were taking for diabetes. For example, a highly educated respondent said, “Every day I use insulin injection two times before meal to control my sugar levels after heavy meals. I also take metformin 850 mg twice daily in the morning and evening (M5_44 years)”.

Participants reported to have knowledge regarding the importance of medications. All participants agreed that taking medicines regularly as prescribed by their physicians is essential to control diabetes and prevent complications. None of the participants, however, were aware of the side effects of the medicines. A participant reported that, “My doctor only told me how many times to take the medicines and the pharmacist gave me all the medicines after I paid for them, but nobody ever informed me about the side effects of the medicines and what I should do if I have any problem (F7_60 years).”

### Experiences of adverse effects of medication

Most of the participants indicated that they did not suffer from any severe adverse effect from their medications, although a few of them stated that they suffered from minor health problems that they think are due to their medications. One of the male respondents mentioned, “I suffered from different physical problem for example hair loss, burning in the body and generalized illness. I don’t know if my diabetes medications were the cause for these problems. (M4_63 years)”. Another participant reported that, “First when I took my diabetes medicines, I felt dizziness and was afraid. My husband told me to take some sweet dish and rest. I was fine after that and had no problems (F2_38 years).”

A few participants mentioned that they had some problems with digestion, constipation, and occasional diarrhea which might be attributable to diabetic medications. However, most of the participants indicated that they, by and large, considered their medication as safe.

### Medication adherence

Patients were asked about what problems they encountered in taking all their medications as prescribed by their doctors. All except two participants mentioned that they do often forget to take all their medications on time. In short, forgetfulness was the major reason for not taking a medication on time. A few participants mentioned that they sometimes forget to take all their medications on time when they feel bad. Two participants mentioned that they purposely missed their medications when they were in good health. One participant said, “In the morning, I am very busy to prepare my children for school, prepare food, get ready and rush for office. I often forget to take my morning medicines and skip my breakfast (M3_48 years).”

It is important to note that none of the participants unilaterally altered or adjusted their medications or dose without consultation with physicians. However, in case of vitamins, dietary supplements and medications for lipids, a few participants took a more liberal approach and ignored physicians’ suggestions as these were not deemed necessary. One participants said, “My doctor prescribed me with six different medicines, but only one for diabetes. I don’t need the vitamins and cholesterol lowering drugs all the time as I eat balanced diet and it is difficult to take so many medicines (M4_63 years).”

In the absence of any medical insurance or government program for social support, the cost of medicine accounted for the largest component of the health care expenditure for all participants. Not surprisingly, cost of medications imposed too much of a burden for many participants and their families. In many cases, participants often depended on financial assistance from other family members to pay for medications. For example, one of the female respondents mentioned, “My son lives and works abroad, and he pays for all my medicines. Often when the money comes late I have to wait to buy my medicines (F7_60 years).”

All the participants were satisfied with the support they received from the family members and friends. Most of the participants felt that they were self-dependent in terms of remembering their medications and doses and did not seek any external support. However, a number of the respondents expressed their difficulties attending regular or monthly check-ups with their physicians, that were not often deemed necessary by other family members. As a result, respondents often failed to maintain the regular check-up with their physicians.

## Discussion

This study explored the patients’ perspective of type 2 diabetes and medication adherence in an urban area in Bangladesh. Given the paucity of research on diabetes in Bangladesh, this is a pioneering study especially since it is a qualitative study aimed at better understanding the unique perspective of patients with type 2 diabetes, its nature and impact and the need for adherence to medications.

This study showed that participants had knowledge about the cause of diabetes. A previous study conducted in Bangladesh on patients with type 2 diabetes reported similar results [25]. The study also revealed that while patients knew that they had diabetes, they were unsure about the type of diabetes that they have, which is congruent with a previous study in Bangladesh [25]. Patients’ lack of knowledge on diabetes care can impede their ability to control the disease. However, a number of participants had serious misunderstandings about mild diabetes that they considered as passing of sugar in urine but not requiring any medical treatment. Such misconceptions might lead to negligence of care and poor health outcomes. A mixed understanding of diabetes and its risk factors had been identified in a number of other studies [26–28].

Diabetes is a lifelong chronic disease and lifestyle modifications are vital for optimum management of the disease. Most of our respondents were aware about the importance of diet, medications and physical activities for controlling diabetes. However, adherence to dietary plans and physical activity was not followed by most of the participants. A previous study in Bangladesh also reported similar findings [29]. However, the study shows non-adherence to physical activity and healthy lifestyle among the participants which needs to be explored further. Previous studies among patients with diabetes in Bangladesh and among migrant Bangladeshis in the UK also identified poor understanding among patients on the dynamics of diabetes and healthy lifestyle [15, 30].

Despite receiving treatment in a specialized diabetes center, participants had misconceptions about medication use. Absence of proper knowledge on medication, often causes delay in seeking treatment, resulting in long term ill health, having serious impact on the individual, the family and in turn, on the society at large. However, once medications were initiated, the participants became aware about the importance to continue the medications for their wellbeing. The participants perceived that medications help control the blood sugar level and eventually diabetes. However, a number of factors often act as barriers in preventing them from regularly taking their prescribed medications.

Diabetes impacts on social life, creates psychological stress, and degrades overall quality of life [31, 32]. Lack of freedom to eat rice, fruits and sweets impair normal lives of people with diabetes. Our study shows that dietary restrictions might create psychological stress on the patients which, subsequently, might lead to depression. A number of previous studies carried out in Bangladesh also corroborated this important finding of the study and reported an association between diabetes and depression [33, 34], which might be explained by this study. The study participants also noted that they received inadequate support from health care providers regarding dietary, discipline and physical activity.

Most of the respondents did not report any serious adverse effects of diabetes medications. A tendency of taking regular medications was observed. However, family support seems to have an important role in this regard [35]. Nevertheless, most of them acted as responsible parties in taking their medications. Participants also reported adequate medication adherence. Contrastingly, a systematic review revealed that treatment adherence is poor with the patients taking insulin [36]. The contrast with our study could be attributed to small number of study participants using insulin. However, there are few studies that assessed patients’ understanding of diabetes and medication adherence, and thus it is difficult to compare our results with those of others. Moreover, there is a need to provide better health education about diabetes and its management in Bangladesh [37, 38]. A recent study reported that patients with diabetes were willing to pay a small amount of money for receiving text messages related to diabetes [39]. Using innovative information technology and mobile phone text messaging might be useful tools for further promoting medication adherence and diabetes management in Bangladesh [40, 41].

This study suggests that there is limited understanding of diabetes, its management and barriers to lifestyle modification in Bangladesh. Unfortunately, Bangladesh health system offers only limited treatment and care services for people with diabetes. A review of health literature also underscores the scarcity of health care services for patients with diabetes [42, 43]. At BIHS hospital, first consultation with the physician as well as some of the basic treatment related services are free. However, patients find the cost of subsequent visits and treatment to be high and that some senior citizens have to depend on their family for financial support of their diabetes treatment.

This is a qualitative study with a small number of patients visiting a tertiary hospital in Dhaka city, Bangladesh, with ready access to specialized physicians and dieticians. The study findings, therefore, might not be applicable to other settings in Bangladesh where healthcare services for diabetes are much limited. Our respondents were interviewed in a hospital setting which might have some influence on the respondents. In addition, the study was not aimed at collecting data about health care providers’ perspective on medication adherence which could have provided a more holistic view on the problem. Moreover, although participants were invited to provide feedback on the findings none complied with such request.

## Conclusions

This study identified a number of key themes that could be useful in designing interventions aimed at further enhancing diabetes knowledge, self-management and medication adherence among patients with type 2 diabetes in Bangladesh.

Healthcare providers should explore to better understand patients’ perspective on diabetes, medication beliefs, identify psychological stress and provide more effective health education interventions to enhance medication adherence.
